# The Military Exhibition

**Published:** 1890-06-07

**Authors:** 


### The Military Exhibition.

The other day we had a spare hour or two and thought that a walk round what *Punch* calls the "Sodgeries" would do us good. We hadn't been long in the exhibition before we recognized some familiar figures at the various stalls devoted to the needs and demands of the healing art. First in the row was that neat temple of invalid gastronomy kept by the noted firm of Brand. Here were to be seen concentrated essences, soups, savoury preparations to tempt the sick one's wayward appetite, with an infinite variety of changing food. We noted besides various biscuits—savoury, digestive, and some called "campaign"—which seemed to be a useful and pleasant form of condensed food stuff.

Next came those well-known artificial limb makers, Ferris and Co., of Great Russell Street. We have so recently described their manufactures that we must content ourselves with noticing a good belt for supporting an artificial leg, the principle of which is that of pulley suspension. It quite does away with the old cumbrous and embarrassing brace. Also on view was a very light crutch, the spring of which was due to the elasticity of the two sides of the fork. When pressure is applied these bow out, on being relieved from it the crutch resumes its natural length. The firm has a good exhibition of the limbs, showing the compound lateral movement ankle joint and the other well-known features of the specialities.

J. Weiss and Co. have a splendid case of surgical instruments on view. Amongst them we specially noted a very complete army surgeon's case and a general amputating case. A pretty thermometer, for suspension from a centre cord in a room, has radiating indices so arranged that from whatever point of view the instrument may be looked at, the height of the fluid can always be noted.

Next come the exhibits of Evans, Wormull, and Co. This firm has a large department at their stall of ambulance appliances, boxes containing fracture and wound appliances, fitted stretchers, waterproof specialities, sheets, beds, baths, bottles, and all kinds of india-rubber goods. They have also a very good collection of the ordinary surgical instruments on view.

On the same side are the beautifully made trusses and artificial limbs of Salmon and Ody. Here one can see the mechanism of their truss, and note how carefully every part is fitted: The metal work is nickled, thus doing away with the old trouble of a rusty steel spring rubbing through the leather and iron-moulding one's clothes.

Then appears the glittering display of Arnold and Son, of Smithfield. In the centre is a gorgeously fitted general surgeon's case, probably for presentation. We note also an extremely complete eye operation case, which makes us rather inclined to trespass on the precepts of the tenth commandment. There are many beautifully made and very portable electrical apparatus. We can remember that when clerking under a physician who was fond of using electricity, the ward battery weighed over a hundred-weight, and required a sort of perambulator to move it. Here we see the same amount of electrical power contained in a neat hand battery. Augove's accident case is also on view. We can speak favourably of this, especially when fitted as an obstetrical case. Ours travelled many hundreds of miles on horseback with us.

Richardson and Co., of Leicester, have a fine display of medicines and cases for the same ranging from an emergency case at half-a-guinea up to a fitted cabinet for a doctor's surgery, costing, we should suppose, when filled, at least £50. There are also volunteer medicine cases, a first aid case, and others to be deposited at centres where they can be used for any sudden accident.

The last, but not least in our estimation of the stalls visited was the invalid food exhibit of Mason and Co. Here we notice beautifully prepared essence, bouillon, and concentrated forms of beef. We have recently annotated on these in our columns, and so shall add nothing to our before favourably expressed opinion. Besides the various beef extracts there are savoury and nourishing forms of soups and an excellent sauce, the O. K.

We hope to return shortly, and describe the ambulance annex and other exhibits which are of interest to the medical and nursing world.

